# The usefulness and utilization of Gold-finger retractor for endoscopic thyroid surgery

**DOI:** 10.3389/fendo.2023.1228657

**Published:** 2023-09-19

**Authors:** Jian Ruan, Bin Dai, Jian Guo Zhao, Long Tao, Fan He

**Affiliations:** ^1^ Department of Thyroid and Breast Surgery, Wuhan No. 1 Hospital, Wuhan, China; ^2^ Department of Hepatobiliary Surgery, Wuhan No. 1 Hospital, Wuhan, China

**Keywords:** papillary thyroid carcinoma, endoscopic surgery, TOETVA, Gold-finger retractor, clinical application

## Abstract

**Aims:**

In endoscopic surgery, the visual field is frequently obstructed by muscles, blood, and even smoke. To overcome this problem, we have developed a new detachable Gold-finger retractor for narrow-space surgery.

**Methods:**

Gold-finger retractor was used in 30 patients to facilitate surgical field exposure and smoke discharge, while in 27 patients, percutaneous silk thread suspension was employed for the same purpose. Both groups underwent endoscopic unilateral thyroidectomy and unilateral central lymph node dissection via oral vestibular microincision combined with the axillary-assisted approach. A comparative analysis was conducted to evaluate the efficacy of the Gold-finger retractor and silk thread suspension in relation to intraoperative exposure effect, surgical fluency, surgeon’s comfort, operation time, postoperative complications, and length of hospital stay. This analysis was based on surgical video recordings and postoperative indicators.

**Results:**

With Gold-finger retractor support, surgeons were able to perform meticulous operations. Complication rates were similar between the two groups, and no serious complications occurred. The number of lymph nodes dissected in the Gold-finger group was significantly greater than that in the routine group (12.43 ± 6.18 and 5.7 ± 2.95, respectively). Further analysis of surgeons’ comfort (visibility and convenience in peeling) revealed that the Gold-finger group was significantly better. Electrosurgery smoke was removed effectively with Gold-finger, and the operation time was significantly reduced.

**Conclusion:**

In thyroid surgery, Gold-fingers enhance visual field resolution, avoid muscle cutting, save time, and improve the surgical experience.

## 1 Introduction

Papillary thyroid carcinoma is a primary malignancy derived from thyroid follicular cells. It is the most prevalent form of differentiated thyroid cancer worldwide (1, 2), particularly among younger women. Aesthetic outcomes of thyroid surgery, especially the cosmetic effects of postoperative neck scars, are a key concern for these patients (3). Open thyroidectomy has been the standard procedure for thyroid cancer for a long time, but neck scars are considered a stigma that can affect the quality of life. Various minimally invasive thyroidectomy techniques, including endoscopy and robotic thyroidectomy, have been developed to improve cosmetic outcomes, and some of these techniques are scar free. Endoscopic thyroidectomy is more suited for resource-limited settings due to the high cost and equipment requirements for robotic surgery. In endoscopic surgery, the surgical site can be accessed from other areas of the body, such as the axilla or breast, thus avoiding any scar in the exposed parts of the body (4–6).

Natural oral transendoscopic surgical thyroidectomy is a new way to avoid surgical scars (7, 8). Since 2008, natural orifice transluminal endoscopic surgical techniques via the oral route for thyroidectomy have been developed, and several studies have demonstrated the safety and effectiveness of this approach (1, 7, 9, 10). However, a relatively high incidence of postoperative psychiatric neurological injury (mental nerve

Injury,MNI) is being noticed, which in some studies was as high as 100% (11). In some cases, numbness can last even 3-12 months (12). Therefore, we developed a modified method, the axillary channel-assisted transoral endoscopic vestibular access (ACA-TOETVA), which can reduce trauma related to vestibular access besides preserving the cosmetic value of the scarless neck offered by TOETVA (13).

Although Transoral Endoscopic Thyroidectomy Vestibular Approach (TOETVA) has been widely recognized, it also poses intraoperative challenges because of occasional visual field obstruction caused by muscles and blood vessels. In addition, due to the nature of minimally invasive surgery, the limited view exaggerates the negative impact of surgical plumes shielding the surgical field. Thus, in thyroid endoscopic surgery with narrow working space, there is a need for special instruments that can be used to manipulate muscles or blood vessels and even exhaust smoke at the same time. To address this need, we designed a new type of retractor (Gold-finger retractor) for providing an additional degree of flexibility and convenience to endoscopic surgeons.

In this work, we report the clinical application of the Gold-finger retractor in endoscopic thyroidectomy and present our initial experience.

## 2 Materials and methods

### 2.1 Gold-finger retractor

**Figure 1:**
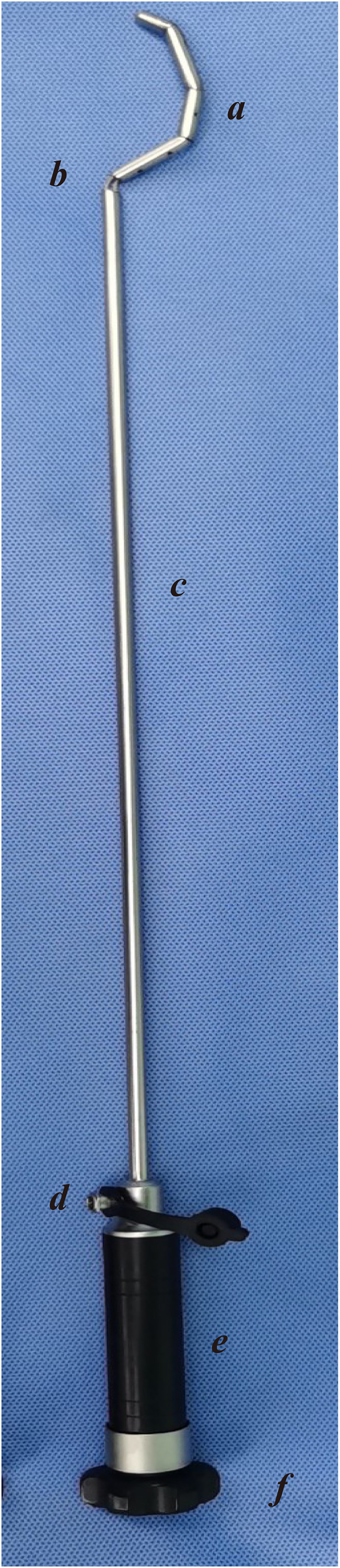
Photograph of the Gold-finger retractor. The Gold-finger retractor comprises three components, namely the control handle, insertion rod, and the distraction section. With the detachable design, it is easy to change the retained position to any arbitrary location on demand. **(A)** Deformable hook head; **(B)** Built-in steel wire; **(C)** Connecting rod; **(D)** Smoking hole; **(E)** Control handle; **(F)** Control runner.

**Figure 2:**
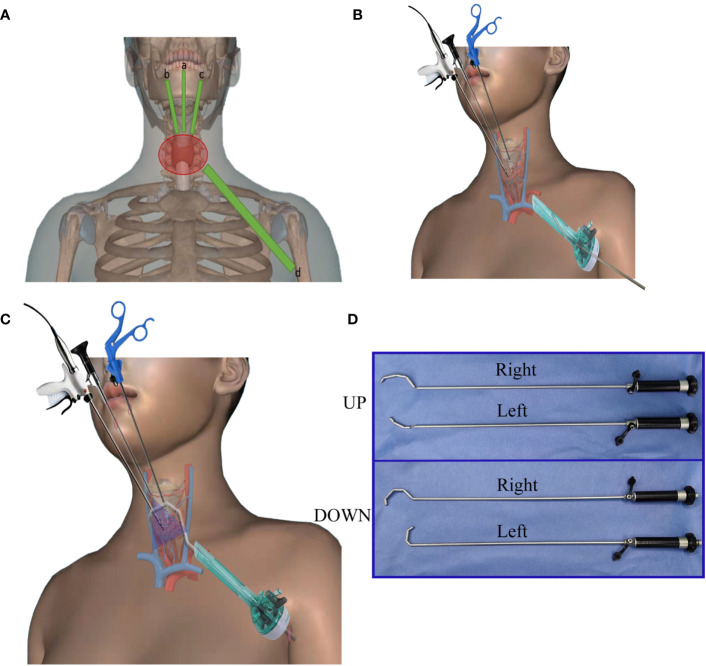
Schematic diagram of the use of Gold-finger. **(A)** 5 mm **(A–C)** and 10 mm **(D)** trocar positions of oral vestibule and axillary channels. **(B)** Relaxed state hook head can easily pass through the 10 mm trocar. **(C)** Once within the designated operating space in the neck, the joints located at the front end of the retractor are tightened, and the vent on the control handle is connected to negative pressure suction to assist exposure and smoking. **(D)** Gold-finger retractor in different states; relaxed state (UP), working state (DOWN).

The Gold-finger retractor comprises three components, namely the control handle, the insertion rod, and the distraction section. The anterior spreading section consists of 3-4 hollow cylinders, with a steel wire located at the center of the cavity. The steel wire is linked to the upper end of the initial cylinder section and a control handle, allowing for the adjustment of the tightness of the connection between the cylinder sections in the spreading section. Once the cylinders are fully tightened, they can be integrated into a cohesive unit and seamlessly connected to form various shapes. The cylinders are equipped with multiple small holes, which, when connected to negative pressure suction through the control handle vent, generate negative pressure at the front-end small holes via the overall hollow design, effectively removing surrounding smoke (**Figures 1**, **2**).

Upon relaxation of the distraction segment, it may be introduced through the 5 mm trocar and promptly secured upon entry into the subcutaneous space of the neck. All incisions in the axillary region were exclusively planned for the left side, with the first assistant positioned on the left of the surgeon, thereby enabling the surgeon to deftly manipulate both the 5 mm endoscope and the Gold-finger. Two distinct Gold-fingers were devised to facilitate exposure of the surgical field during left and right thyroidectomy procedures, respectively.

### 2.2 Directions for using the Gold-finger retractor

**Figure 3:**
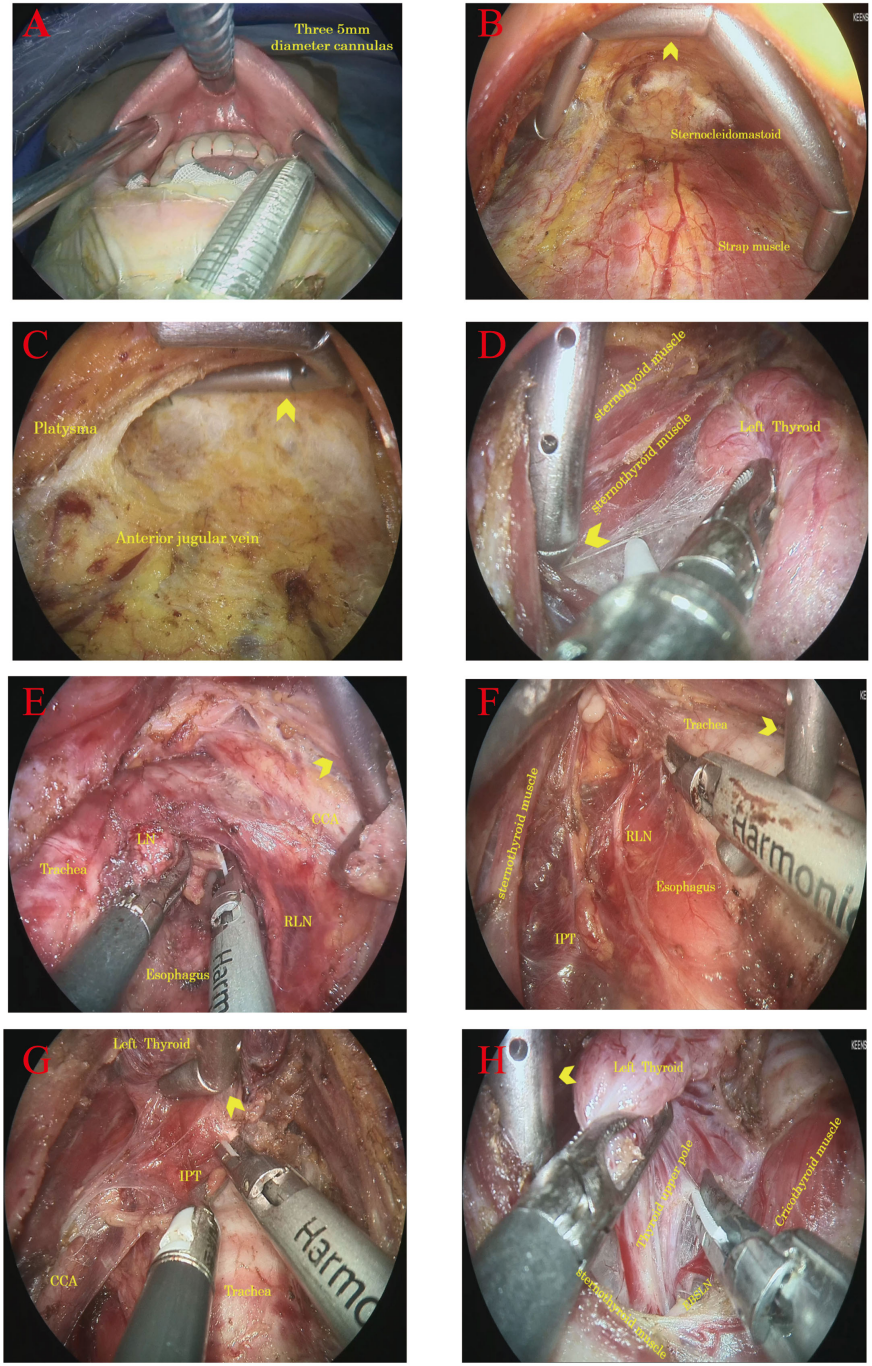
Intraoperative photographs showing the function and application details of Gold-finger. **(A)** 5 mm trocar positions of oral vestibule; **(B)** Gold-finger retractor to assist horizontal jacking flap, expand the operation space; **(C)** Gold-finger retractor longitudinal jacking flap, to prevent collapse first neck horizontal stripes; **(D)** Gold-finger retractor to help open the thyroid capsule; **(E)** Gold-finger retractor used to push the common carotid artery and help clean the lymph nodes in the central region; **(F)** The Gold-finger retractor helped clean the lymph nodes in the central region by pushing them back into the trachea. **(G)** The Gold-finger hook assists *in situ* retention of the parathyroid gland by hooking the thyroid gland. **(H)** Gold-finger retractor helped expose the upper pole of the thyroid and external branch of the superior laryngeal nerve.

Upon insertion into the axillary-neck channel, the front end of the Gold-finger retractor assumes a relaxed state through the 10 mm trocar. Once within the designated operating space in the neck, the joints located at the front end of the retractor are tightened, and the vent on the control handle is connected to negative pressure suction (**Figure 2**). A comprehensive depiction of the functionality and application of Gold-finger is presented in **Figure 3**.

### 2.3 Study population and data collection

The present study was approved by the Ethics Committee of Wuhan No. 1 Hospital. The study population comprised 57 patients who were hospitalized in our department between February 2022 and February 2023 with a diagnosis of thyroid cancer. Starting from August 2022, the ACA-TOETVA procedure was performed at our center using the Gold-finger retractor (Hangzhou Tonglu Medical Instrument Equipment Co. Ltd., China), which was designed by the first author. The patients were stratified into two groups based on whether or not the Gold-finger retractor was employed during the surgical intervention.

During the operation, the Gold-finger retractor was used to facilitate surgical field exposure and smoke discharge in 30 patients (group A), while percutaneous silk thread suspension was used for the same purpose in 27 patients (group B). Both groups underwent endoscopic unilateral thyroidectomy and unilateral central lymph node dissection via oral vestibular microincision combined with an axillary-assisted approach.

A comparative analysis was conducted to evaluate the efficacy of the Gold-finger retractor and silk thread suspension in relation to intraoperative exposure, surgical fluency, surgeon’s comfort (14), operation time, postoperative complications, and length of hospital stay. This analysis was based on surgical video recordings and postoperative indicators.

### 2.4 Surgical technique

The details of the surgical procedure are described in our previous article (13).

### 2.5 Statistical analysis

Statistical analysis was performed using GraphPad Prism 8.0.2. Continuous variables were expressed as mean ± standard deviation and the between-group differences were assessed using an independent sample *t*-test. The statistical significance of sex differences was analyzed using the Chi-square test. *p*-values of less than 0.05 were considered as being statistically significant.

## 3 Results

The baseline data of patients are shown in **Table 1**. Women accounted for the majority of patients in both groups. All the patients had stage T1 disease, and there was no significant between-group difference regarding age, body mass index (BMI), thyroiditis, thyroid size, and weight. Operative outcomes and complications are presented in **Table 2**. The mean operation time in group A and group B was 90.17 minutes and 115.2 minutes, respectively. The incidence of complications was not significantly different between the two groups. No major complications were recorded. However, the number of dissected lymph nodes in group A was significantly greater than that in group B (12.43 ± 6.18 vs 5.7 ± 2.95), which may be attributed to the fact that the Gold-finger enables better endoscopic visual access, enabling surgeons to perform delicate and meticulous surgery while preserving the recurrent laryngeal nerve, normal parathyroid glands, and superior laryngeal nerve branches. Average surgeon comfort was calculated for both visibility and ease of dissection and was found to be significantly better in group A (**Table 3**).

**Table 1:** Comparison of the clinical and pathological characteristics of patients in the two groups.

Variables	Group A	Group B	*p*-value
Male: Female	2:28	3:24	0.66
Age (years)	37.30 ± 10.77	32.93 ± 8.70	0.1
Body mass index	22.05 ± 3.86	21.96 ± 3.41	0.92
Tumor size (cm)	0.78 ± 0.44	0.59 ± 0.37	0.07
Thyroid weight (g)	27.21 ± 6.58	24.63 ± 7.1	0.16
Thyroiditis, Yes : No	14:16	11:16	0.79
TNM clinical stage I	30	27	

**Table 2:** Operative outcomes and complications.

Variables	Group A	Group B	*p*-value
LN metastasis	2.03 ± 2.19	0.74 ± 1.2	0.01
Harvested LN	12.43 ± 6.2	5.7 ± 2.94	<0.0001
Operating time (min)	90.17 ± 9.78	115.2 ± 17.12	<0.0001
Hoarseness	1	2	
Ecchymosis	0	3	
Hematoma	0	0	
Infection	0	0	
Post−operative Length of stay (days)	2.93 ± 1.11	3.42 ± 0.9	0.08

LN, lymph nodes.

**Table 3:** Mean scores for surgeon’s comfort level*.

	Group A N=30	Group B N=27	*p*-value
Visibility	9.73 ± 0.52	7.48 ± 1.01	<0.0001
Ease of dissection	9.48 ± 0.57	7.19 ± 0.88	<0.0001

*Scale: 1-10.

**Figure 4:**
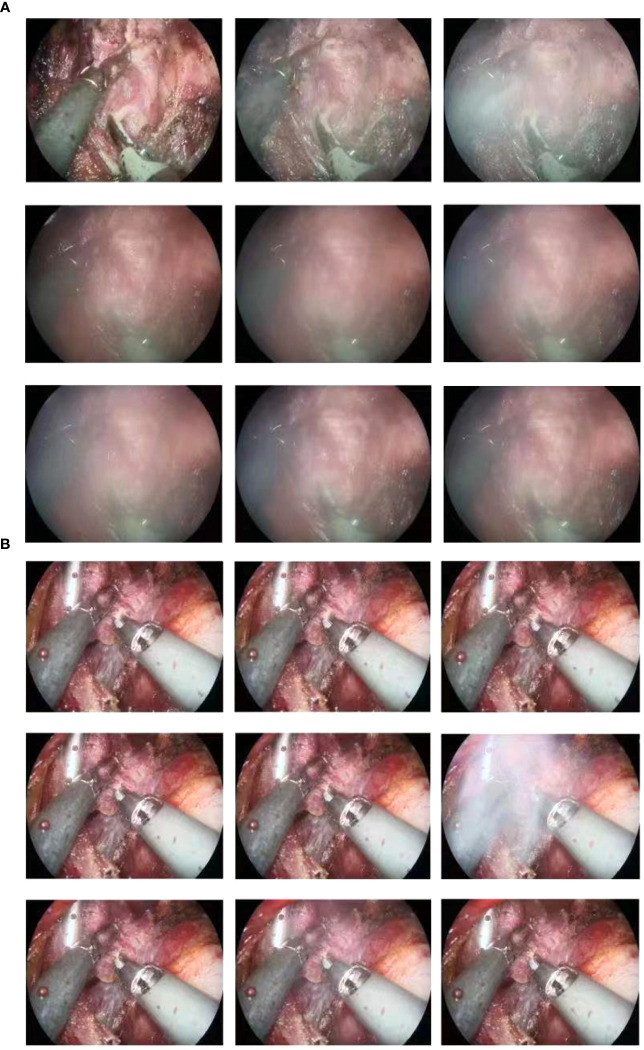
The Gold-finger has good efficacy in removing smoke produced by electrosurgery. **(A)** Production of smoke during electrosurgery without the use of Gold-finger. **(B)** Gold-finger also provides a better surgical field by removing smoke.

Images captured by our surgical video suggest that gold-finger has good results for smoke removal produced by electrosurgery (**Figure 4**), although this aspect was not evaluated using objective criteria.

## 4 Discussion

There has been a significant increase in the prevalence of thyroid cancer, particularly among women (15–18). Most patients with thyroid cancer undergo thyroidectomy. The aesthetic outcome of the surgical procedure is a paramount concern among patients. With the advent of endoscopic surgery, the presence of any discernible scarring on the neck after thyroidectomy can be circumvented. In recent years, various treatment methods have been developed for thyroid diseases; however, most of these methods result in visible scarring in areas other than the anterior neck region. Transoral endoscopic thyroidectomy (TOET) is the only surgical procedure that can be considered truly scarless (19). Among the different approaches for TOET, the TOET vestibular approach (TOETVA) is the most commonly used due to the favorable surgical outcomes and low incidence of complications (13, 20–23). Despite the numerous advantages of TOETVA, it may lead to complications such as postoperative mental nerve injury, which cannot be entirely prevented despite due diligence on the part of the surgeon. To address this issue, a modified approach, known as the axillary channel-assisted transoral endoscopic thyroidectomy vestibular approach (ACA-TOETVA), was developed. In our previous study, ACA-TOETVA was found to mitigate the supplementary trauma incurred during workspace establishment and specimen extraction by generating supplementary axillary neck channels, thereby providing superior protection for the mental and mental nerves (13). Furthermore, the use of auxiliary devices reduces the operation time and intraoperative bleeding and affords greater convenience in endoscopic operations (13). Nevertheless, certain challenges persist in clinical practice, including limited working space and obstructed vision due to smoke.

The production of smoke during electrosurgery is a major stressor for surgeons, which can extend operation duration and compromise surgical quality (24, 25). While conventional surgical attractors offer advantages in smoke elimination, their suction strength poses a challenge in achieving a balance between effective smoke removal and preservation of the surgical field. The judicious application of the Gold-finger not only eliminates smoke but also enhances the surgical field, a critical factor in ensuring surgical safety (field of view continuity is guaranteed, reducing the time required for instrument exchange).

In this study, all patients underwent central lymph node dissection. Meticulous central lymph node dissection is a challenge in endoscopic surgery due to the two-dimensional operational view and the difficulty of operating nonflexible instruments (26). In the absence of Gold-finger, we had to use silk traction to retract the strap muscle for central lymph node dissection. However, there are several drawbacks associated with the use of silk thread in surgical procedures. Firstly, it has the potential to cause cut injury to the muscle, which may result in bleeding. Secondly, the silk thread is not easily able to pull the innermost band muscle, and its exposure effect is limited, particularly in cases where the upper pole of the thyroid is hypertrophied. This can make the treatment of the upper pole more challenging. Thirdly, the needle must be passed through the skin and the band muscle before being fixed outside the skin, which can add 5-10 minutes to the procedure in order to achieve the desired traction effect. The generation of acting force in the silk thread and needle hook is relatively singular in both direction and manner, with limited adjustability in terms of acting position, rendering it unsuitable for the changing surgical scene. The use of the needle and hook in puncturing the flap poses a risk of collateral damage, as does the placement and withdrawal of the hook in the cavity. On the contrary, the Gold-finger can be rotated and positioned within the cavity without causing any adverse effects. This attribute enables the Gold-finger to adapt to varying surgical scenarios and perform a range of auxiliary functions. Following the utilization of the Gold-finger, the common carotid artery is pushed, the space around the recurrent laryngeal nerve is enlarged, and it is easier to clean the lymph nodes behind the common carotid artery and the recurrent laryngeal nerve. During the process of cleansing the lymph nodes in the central region, the gold fingers are utilized to mobilize the glands in an upward direction, thereby enabling the left hand to employ the fine dissecting forceps for meticulous dissection, allowing for complete exposure of the recurrent laryngeal nerve from all angles. This facilitates a comprehensive dissection of the central lymph nodes. Thus a greater number of lymph nodes can be removed, although many of these are not necessarily positive lymph nodes. There are, however, other possible explanations. In group A, a notable prevalence of severe Hashimoto’s thyroiditis was observed, resulting in a significant increase in the number of lymph nodes within the central area. Specifically, one case exhibited 35 lymph nodes, while another case displayed 20 lymph nodes, thereby contributing to the higher overall average value in group A.

In this study, differences in comfort levels were documented between the two groups of surgeons. The observed comfort score indicated a significant enhancement in surgeon comfort with the use of Gold-finger. Furthermore, no significant injuries or adverse effects were reported, suggesting the safety of Gold-finger.

Some limitations of this study should be acknowledged, including the small sample size and the absence of a validated comfort scale. Furthermore, the study did not report oncological outcomes due to the short follow-up period. Larger studies with long-term follow-up will provide more robust evidence. Additionally, the study lacked objective criteria for the evaluation of smoke removal effects.

In conclusion, Gold-finger affords better visual field exposure, avoids muscle cutting, saves time, and improves the surgical experience in thyroid surgery. Gold-finger can be considered as simple robot arms, as described below.

First, when the subcutaneous space of the neck is not completely established, the Gold-finger is placed close to the flap to lift it upward and prevent it from collapsing, especially the flap at the first cervical transverse crease. Second, when opening the gap between the thyroid and the strap muscle, the tip of the Gold-finger is used to push the muscle outward and sideways to assist in separation and exposure. Third, while dealing with the superior pole of the thyroid gland, the distal segment of Gold-finger is used to penetrate the outer side of the highest point of the upper pole, and the strap muscle is effectively pushed away. This can enable complete exposure of the blood vessels of the upper pole of the thyroid gland and is advantageous for the visualization and preservation of the external branches of the superior laryngeal nerve. As a fourth consideration, during the dissection of the central lymph nodes, it is recommended to initially preserve the layer of fascia tissue separating the recurrent laryngeal nerve and the common carotid artery. Subsequently, the common carotid artery may be displaced outwardly using a Gold-finger or the trachea may be displaced inwardly using a Gold-finger. This technique serves to increase the distance between the recurrent laryngeal nerve and the trachea and Berry’s ligament, thereby facilitating the dissection process. Gold-finger can be used to elevate the thyroid gland and lymphatic adipose tissue in an upward direction, allowing for the left hand to be released, enabling the utilization of fine separation forceps to complete the operation meticulously, thereby promoting the preservation of the lower parathyroid gland in situ.

Additionally, in the event of intraoperative vascular bleeding, the distal end of Gold-finger can be employed to apply pressure to the blood vessel, temporarily controlling the bleeding, preventing contamination of the surgical field, and allowing for subsequent hemostasis procedures to be carried out in a timely manner.

## Data availability statement

The raw data supporting the conclusions of this article will be made available by the authors, without undue reservation.

## Ethics statement

The studies involving humans were approved by Ethics Committee of Wuhan No. 1 Hospital. The studies were conducted in accordance with the local legislation and institutional requirements. The participants provided their written informed consent to participate in this study.

## Author contributions

JR, BD, and JZ contributed to the conception and design of the study. JR organized the database. LT performed the statistical analysis. BD wrote the first draft of the manuscript. JR, JZ, and FH wrote sections of the manuscript. All authors contributed to the article and approved the submitted version.

## References

[1] Liu Z Li Y Wang Y Xiang C Yu X Zhang M. Comparison of the transoral endoscopic thyroidectomy vestibular approach and open thyroidectomy: A propensity score-matched analysis of surgical outcomes and safety in the treatment of papillary thyroid carcinoma. Surgery (2021) 170(6):1680–6. doi: 10.1016/j.surg.2021.06.032 34284897

[2] Lam AK. Papillary thyroid carcinoma: current position in epidemiology, genomics, and classification. Methods Mol Biol (2022) 2534:1–15. doi: 10.1007/978-1-0716-2505-7_1 35670964

[3] Kim K Lee S Bae JS Kim JS. Comparison of long-term surgical outcome between transaxillary endoscopic and conventional open thyroidectomy in patients with differentiated thyroid carcinoma: A propensity score matching study. Surg Endosc (2021) 35(6):2855–61. doi: 10.1007/s00464-020-07721-2 32556767

[4] Ikeda Y Takami H Niimi M Kan S Sasaki Y Takayama J. Endoscopic thyroidectomy by the axillary approach. Surg Endosc (2001) 15(11):1362–4. doi: 10.1007/s004640080139 11727158

[5] Wang C Feng Z Li J Yang W Zhai H Choi N. Endoscopic thyroidectomy via areola approach: summary of 1,250 cases in a single institution. Surg Endosc (2015) 29(1):192–201. doi: 10.1007/s00464-014-3658-8 24986013

[6] Park JO Kim SY Chun BJ Joo YH Cho KJ Park YH. Endoscope-assisted facelift thyroid surgery: an initial experience using a new endoscopic technique. Surg Endosc (2015) 29(6):1469–75. doi: 10.1007/s00464-014-3826-x 25159657

[7] Anuwong A Ketwong K Jitpratoom P Sasanakietkul T Duh QY. Safety and outcomes of the transoral endoscopic thyroidectomy vestibular approach. JAMA Surg (2018) 153(1):21–7. doi: 10.1001/jamasurg.2017.3366 PMC5833624 28877292

[8] Wang Y Zhou S Liu X Rui S Li Z Zhu J. Transoral endoscopic thyroidectomy vestibular approach vs conventional open thyroidectomy: meta-analysis. Head Neck (2021) 43(1):345–53. doi: 10.1002/hed.26486 33043571

[9] Zheng G Ma C Sun H Wu G Guo Y Wu G. Safety and surgical outcomes of transoral endoscopic thyroidectomy vestibular approach for papillary thyroid cancer: A two-centre study. Eur J Surg Oncol (2021) 47(6):1346–51. doi: 10.1016/j.ejso.2021.01.028 33558121

[10] Van Den Heede K Brusselaers N Gaujoux S Menegaux F Chereau N. Feasibility and safety of ambulatory transoral endoscopic thyroidectomy via vestibular approach (Toetva). World J Surg (2022) 46(11):2678–86. doi: 10.1007/s00268-022-06666-y PMC9295883 35854011

[11] Tae K Lee DW Bang HS Ahn YH Park JH Kim DS. Sensory change in the chin and neck after transoral thyroidectomy: prospective study of mental nerve injury. Head Neck (2020) 42(11):3111–7. doi: 10.1002/hed.26351 32621344

[12] Zhang D Caruso E Sun H Anuwong A Tufano R Materazzi G. Classifying pain in transoral endoscopic thyroidectomy. J Endocrinol Invest (2019) 42(11):1345–51. doi: 10.1007/s40618-019-01071-0 31187465

[13] Ruan J Yang X Zhao JG Tao L Ning XJ He F. Axillary channel-assisted toetva: an effective way to prevent mental nerve from iatrogenic injury? J Minim Access Surg (2022) 18(3):450–8. doi: 10.4103/jmas.jmas_263_21 PMC9306113 35708390

[14] Neogi P Kumar P Kumar S. Low-pressure pneumoperitoneum in laparoscopic cholecystectomy: A randomized controlled trial. Surg Laparosc Endosc Percutan Tech (2020) 30(1):30–4. doi: 10.1097/SLE.0000000000000719 31425453

[15] Wiltshire JJ Drake TM Uttley L Balasubramanian SP. Systematic review of trends in the incidence rates of thyroid cancer. Thyroid (2016) 26(11):1541–52. doi: 10.1089/thy.2016.0100 27571228

[16] Lim H Devesa SS Sosa JA Check D Kitahara CM. Trends in thyroid cancer incidence and mortality in the United States, 1974-2013. JAMA (2017) 317(13):1338–48. doi: 10.1001/jama.2017.2719 PMC8216772 28362912

[17] Liu C Chen T Zeng W Wang S Xiong Y Liu Z. Reevaluating the prognostic significance of male gender for papillary thyroid carcinoma and microcarcinoma: A seer database analysis. Sci Rep (2017) 7(1):11412. doi: 10.1038/s41598-017-11788-8 28900207 PMC5595899

[18] Camenzuli C Schembri Wismayer P Calleja Agius J. Transoral endoscopic thyroidectomy: A systematic review of the practice so far. JSLS (2018) 22(3). doi: 10.4293/JSLS.2018.00026 PMC6158973 30275676

[19] Jongekkasit I Jitpratoom P Sasanakietkul T Anuwong A. Transoral endoscopic thyroidectomy for thyroid cancer. Endocrinol Metab Clin North Am (2019) 48(1):165–80. doi: 10.1016/j.ecl.2018.11.009 30717900

[20] Jitpratoom P Ketwong K Sasanakietkul T Anuwong A. Transoral endoscopic thyroidectomy vestibular approach (Toetva) for graves' Disease: A comparison of surgical results with open thyroidectomy. Gland Surg (2016) 5(6):546–52. doi: 10.21037/gs.2016.11.04 PMC5233830 28149798

[21] Dionigi G Lavazza M Bacuzzi A Inversini D Pappalardo V Tufano RP. Transoral endoscopic thyroidectomy vestibular approach (Toetva): from a to Z. Surg Technol Int (2017) 30:103–12. 28182829

[22] Anuwong A Sasanakietkul T Jitpratoom P Ketwong K Kim HY Dionigi G. Transoral endoscopic thyroidectomy vestibular approach (Toetva): indications, techniques and results. Surg Endosc (2018) 32(1):456–65. doi: 10.1007/s00464-017-5705-8 28717869

[23] Yi JW Yoon SG Kim HS Yu HW Kim SJ Chai YJ. Transoral endoscopic surgery for papillary thyroid carcinoma: initial experiences of a single surgeon in South Korea. Ann Surg Treat Res (2018) 95(2):73–9. doi: 10.4174/astr.2018.95.2.73 PMC6073045 30079323

[24] Mattes D Silajdzic E Mayer M Horn M Scheidbach D Wackernagel W. Surgical smoke management for minimally invasive (Micro)Endoscopy: an experimental study. Surg Endosc (2010) 24(10):2492–501. doi: 10.1007/s00464-010-0991-4 20339874

[25] Barrett WL Garber SM. Surgical smoke: A review of the literature. Is this just a lot of hot air? Surg Endosc (2003) 17(6):979–87. doi: 10.1007/s00464-002-8584-5 12640543

[26] Kim WW Jung JH Park HY. New technique using the snake retractor for complete lymph node dissection in robotic thyroid surgery: initial experiences. Surg Laparosc Endosc Percutan Tech (2013) 23(1):e1–4. doi: 10.1097/SLE.0b013e31826cc1f5 23386162

